# Effect of air temperature on serum 25-hydroxyvitamin D concentrations: A single institutional large-scale study in Korea

**DOI:** 10.1371/journal.pone.0297495

**Published:** 2024-03-29

**Authors:** Kyung Hee Han, Yujin Jeong, Young Ju Suh, Dong Hoon Suh, Kidong Kim, Yong Beom Kim, Jae Hong No

**Affiliations:** 1 Department of Obstetrics and Gynecology, CHA University Ilsan Medical Center, Gyeonggi-do, Republic of Korea; 2 Department of Biostatistics, Korea University College of Medicine, Seoul, Republic of Korea; 3 Department of Biomedical Science, College of Medicine, Inha University, Incheon, Republic of Korea; 4 Department of Obstetrics and Gynecology, Seoul National University Bundang Hospital, Seongnam, Republic of Korea; 5 Department of Obstetrics and Gynecology, Seoul National University College of Medicine, Seoul, Republic of Korea; Kyung Hee University School of Medicine, REPUBLIC OF KOREA

## Abstract

Vitamin D deficiency is a worldwide health issue especially in women. Serum vitamin D concentrations vary depending on the weather. However, the ideal vitamin D supplementation strategy related to weather remains uncertain. We aimed to investigate the relationship between climate factors and serum 25-hydroxy vitamin D [25(OH)D] concentrations. This study included 11,272 women aged 20–79 who visited a health promotion center for annual checkups between January 2013 and December 2015. We reviewed medical records and collected daily meteorological data. We analyzed the association between serum 25(OH)D concentration and climate factors using simple and multiple regression models and then predicted serum 25(OH)D concentration using multiple fractional polynomial models. The median age of the participants was 51 years (20–79 years), and the mean serum 25(OH)D level was 17.4 ± 8.6 ng/mL. The serum 25(OH)D concentration was lower in young women than in older women. The proportions of women with adequate 25(OH)D levels were 14.9% and 47.0% in the age groups 20–29 and 70–79, respectively. The maximum level of predicted log 25(OH)D was found in September, and the minimum was found in January. In multiple regression analysis, age and monthly mean temperature were associated with 25(OH)D concentrations. Serum 25(OH)D level was predicted using the following formula: log (25(OH)D) = 2.144 + 0.009 × age + 0.018 × ((temperature + 12.4)/10)^2^ (*P* < 0.001, adjusted R^2^ = 0.091). Serum 25(OH)D concentrations changed according to air temperature. An adequate strategy for vitamin D supplementation, based on air temperature, is necessary to maintain healthy serum 25(OH)D levels.

## Introduction

Vitamin D deficiency is a widely recognized global health problem. It is estimated that one billion people worldwide have vitamin D insufficiency. Vitamin D plays a crucial role to maintain the serum concentration of calcium and phosphate; and is critical for bone mineral metabolism [1]. Vitamin D deficiency is associated with autoimmune diseases, infections, cardiovascular diseases, neurological disorders, and certain types of cancers [2].

In previous studies, the vitamin D concentration was lower in women than in men, and the proportion of women with vitamin D insufficiency was 64.5% compared with 47.3% in men [3, 4]. According to the National Health and Nutrition Examination Survey, a decrease in vitamin D levels was more prevalent in young women [5]. Vitamin D insufficiency in young women is associated with poor skeletal and reproductive health [6].

The major source of vitamin D is skin epidermal synthesis via ultraviolet B (UVB) radiation, which accounts for 90% of the vitamin D replenishment [7]. Sunlight changes according to the weather throughout the year; thus, vitamin D levels generally depend on climate factors [8]. Among the climate factors, the level of vitamin D is associated with UVB radiation, cloud cover, rain, snow, or air temperature [9, 10]. Increased sunlight exposure, sunny days, or daily air temperature positively correlates with serum vitamin D levels [11]. Vitamin D concentration fluctuates with the season, often showing lower levels during the winter months [12]. In cold winters, reduced sunlight, decreased outdoor activity, and covered clothing can hinder the synthesis of vitamin D in human skin [13]. To maintain optimal vitamin D levels, oral vitamin D supplementation is recommended. However, there are no definitive guidelines for vitamin D supplementation at weather-reflecting doses [14].

Serum vitamin D concentrations vary according to latitude, skin color, and genetic variations [15, 16]. Korea is located in East Asia in the Northern Hemisphere (33–38°N) and has distinct monthly weather. Koreans have similar genetic inheritance patterns and skin colors [3]. Therefore, we investigated the vitamin D concentration of Korean women and developed a predictive model for serum vitamin D levels to establish a supplementation plan based on climate factors.

## Materials and methods

### Ethics statement

The Institutional Review Board of the Seoul National University Bundang Hospital approved this study (IRB No. B-1610-366-101). This was a hospital-based observational retrospective study. Informed consent requirements for the included individuals were waived because of the minimal or negligible risk of this research and the practical challenges of loss of follow-up in the recruited population.

### Patients and inclusion criteria

The anonymous medical data of 21,620 women aged 20–79 years who visited the Health Promotion Center at Seoul National University Bundang Hospital for regular checkups between January 2013 and December 2015 were reviewed retrospectively after the approval (data access period: from September 2016 to September 2017). None of the participants were pregnant or breastfeeding. Of these, 581 women with thyroid, hypothyroid, or adrenal diseases and 9,767 women with osteoporosis who were taking medication were excluded. A total of 11,272 women were included in this study. The age of the women and the day on which the blood sample was taken were recorded.

### Collection of vitamin D and meteorological data

All included individuals underwent blood tests including vitamin D levels, as part of their health checkups during visits to health centers. Data on vitamin D levels were collected through a retrospective review. The concentration of serum 25-hydroxy vitamin D [25(OH)D], a measurable form of vitamin D was detected using a ^125^I RIA kit (DiaSorin, Stillwater, MN, USA) within 24 h of blood sample collection shading the light. All women were classified into four groups based on serum 25(OH)D levels according to the National Institutes of Health criteria: deficiency, < 12 ng/mL; inadequate, between 12 and 20 ng/mL; adequate, > 20 ng/ml and < 50 ng/mL; and high, > 50 ng/mL. One nanogram per milliliter 25(OH)D corresponds to 2.5 nmol/L 25(OH)D [17].

Suwon was the nearest city to most of the patients included in our study. Thus, we collected data on the weather conditions in Suwon. Daily meteorological data, such as sunshine hours, percentage of sunshine, global solar radiation, temperature, and UVB rays were obtained from the Korea Meteorological Administration website [18].

### Statistical analysis

The clinical characteristics of the patients were analyzed using the chi-square test, Fisher’s exact test, or Spearman’s rank correlation test for categorical or ordinal data, and the t-test or analysis of variance test for continuous data. To investigate the association between 25(OH)D levels and seasonal factors, simple and multiple regression analyses were conducted using the SPSS Statistics software (version 21.0; IBM Corporation, Armonk, NY, USA). The predicted 25(OH)D levels were evaluated using a multiple-fractional polynomial model [19]. An internal validation was performed to evaluate the accuracy of the predictive models. We employed a five-fold cross-validation (CV) approach to obtain an unbiased internal assessment of the predictive performance of the model. During the validation process, Pearson’s correlation analysis between the observed and predicted values obtained from the prediction model in each test set was used to evaluate model performance. The closer the estimated correlation coefficient is to 1, the more accurately the model represents the data. R statistical package was used for the prediction model generation and model validation process (version 4.0.5, ‘mfp’ package in http://cran.r-project.org). Statistical significance was defined as a two-sided *P*-value < 0.05.

## Results

### Baseline characteristics

The median age of the participants was 51 years (20–79 years). The mean serum 25(OH)D concentration was 17.41 ± 8.60 ng/mL, corresponding to inadequate vitamin D level. All women were divided into four groups according to their 25(OH)D concentration (Table 1): deficiency, inadequate, adequate, and high. Age, 25(OH)D measurement time, mean temperature, accumulated ultraviolet A (UVA), and maximum UVB exposure were associated with serum 25(OH)D concentrations (all *P* < 0.001). Among the four groups, the mean age of the deficiency group was lower than those of the other groups (*P* < 0.001). The proportion of women with adequate 25(OH)D levels gradually increased with age. There were no significant relationships between 25(OH)D and precipitation, sunshine duration, or solar radiation quantity.

**Table 1:** Baseline characteristics according to vitamin D status.

**25(OH)D level**	**Deficiency (<12 ng/ml)**	**Inadequate (12–20 ng/ml)**	**Adequate (20–50 ng/ml)**	**High (≥50 ng/ml)**	**P**
	**(n = 3,465, 30.7%)**	**(n = 4,240, 37.6%)**	**(n = 3,532, 31.3%)**	**(n = 35, 0.3%)**	
25(OH)D (mean±SD, ng/ml)	9.1±1.9	15.5±2.2	27.5±6.1	56.6±7.8	<0.001
Age (mean±SD, year)	47.7±12.6	49.8±12.4	54.8±12.1	60.7±10.9	<0.001
Age group (n, %)					<0.001
20–29 years	249 (42.3)	251 (42.6)	88 (14.9)	1 (0.2)	
30–39 years	647 (39.0)	655 (39.5)	353 (21.3)	2 (0.1)	
40–49 years	1,113 (39.9)	1,073 (38.4)	605 (21.7)	1 (0.0)	
50–59 years	845 (25.0)	1,328 (39.2)	1,200 (35.4)	13 (0.4)	
60–69 years	407 (20.6)	680 (34.5)	874 (44.3)	10 (0.5)	
70–79 years	204 (23.3)	253 (28.8)	412 (47.0)	8 (0.9)	
Measured time (mean±SD, month)	6.3±3.8	7.3±3.4	7.4±3.2	8.6±2.7	<0.001
Temperature (mean±SD,°C)	10.2±9.8	13.8±10.2	14.9±10.1	15.3±9.1	<0.001
Accumulated UVA (mean±SD, MJ/m^2^)	0.65±0.36	0.69±0.36	0.71±0.36	0.69±0.36	<0.001
Maximum of UVB (mean±SD, W/m^2^)	0.10±0.06	0.11±0.06	0.12±0.06	0.12±0.06	<0.001
Precipitation (mean±SD, mm/day)	6.55±12.96	7.93±15.61	7.83±15.85	7.85±13.77	0.078
Duration of sunshine (mean±SD, hour/day)	6.54±3.84	6.39±3.92	6.44±3.96	6.46±3.80	0.452
Radiation quantity (mean±SD, MJ/m^2^)	11.85±6.17	11.73±6.11	11.84±6.01	11.32±5.89	0.766

^a^Abbreviations: SD, standard deviation; UVA, ultraviolet A; UVB, ultraviolet B

### Association between serum level of 25(OH)D, age, and months

**Fig 1:**
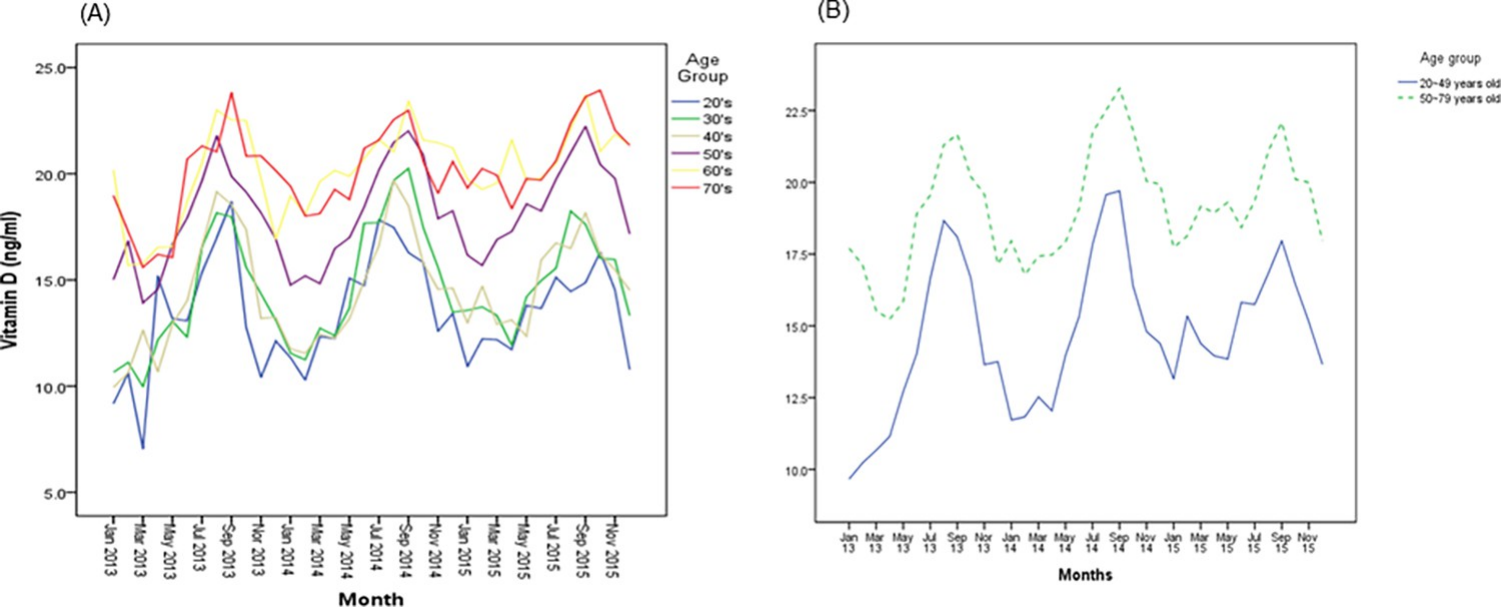
Plots of 25(OH)D according to age groups during the study period. (A, by 10-year age groups; B, based on age 50).

Fig 1(A) shows the serum 25(OH)D levels by 10-year age groups during the study period. Most study participants had low 25(OH)D concentrations (< 20 ng/mL) during the study period. Older age groups tended to have higher 25(OH)D concentrations than younger ones. The lowest 25(OH)D concentration was observed in women aged 20–29 years, whereas the highest was observed in women aged 70–79 years. As shown in Fig 1(B), women aged < 50 years (n = 5,038, 44.7%) had lower 25(OH)D levels than those aged ≥ 50 years (n = 6,234, 55.3%). The peak 25(OH)D concentration in women aged ≥ 50 years occurred later than in women aged < 50 years. The 25(OH)D levels showed a unique monthly pattern in all age groups.

**Fig 2:**
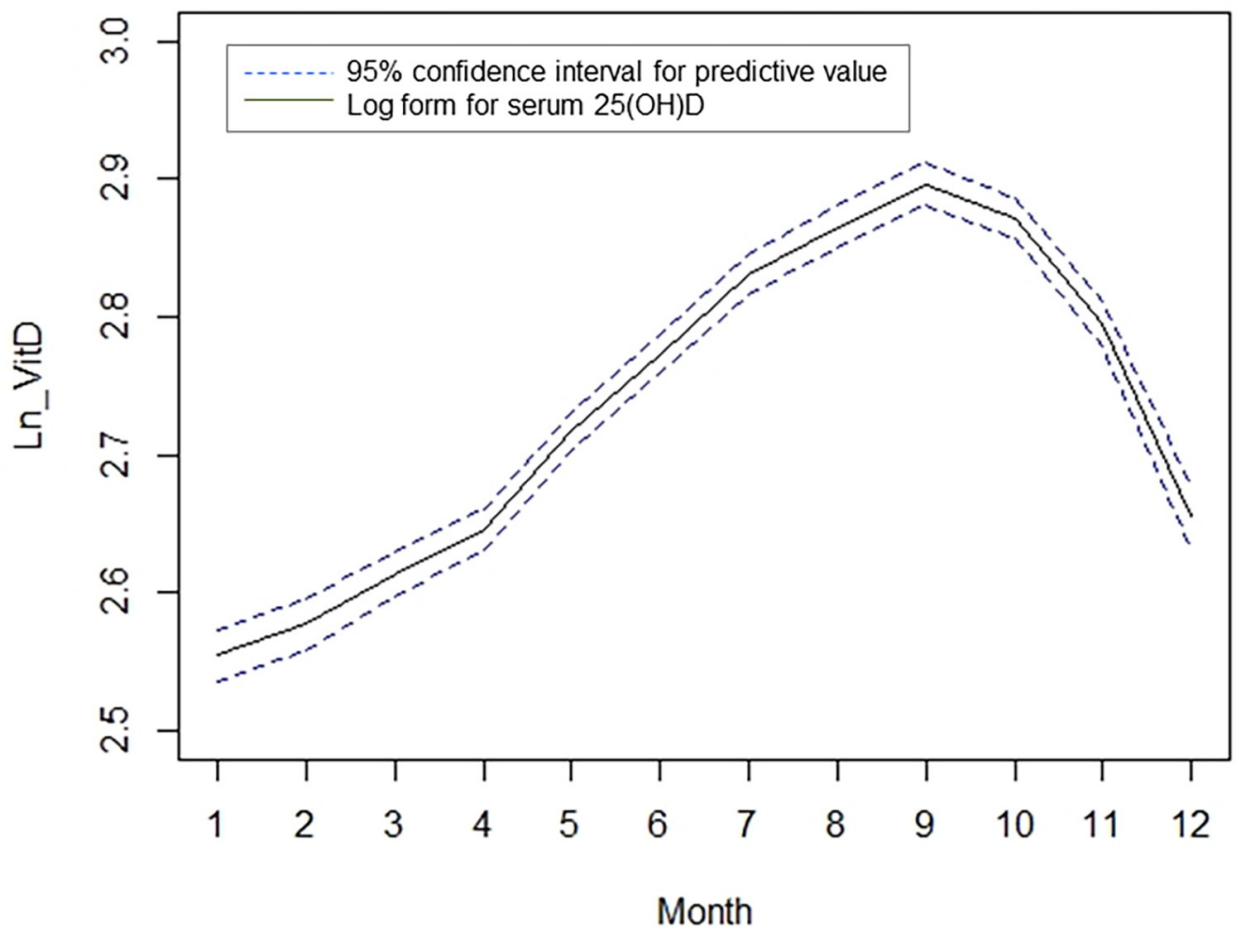
Monthly mean predicted log 25(OH)D value after adjusting for age, measured between January 2013 and December 2015. Black solid line indicates monthly mean predicted log serum vitamin D (25(OH)D, ng/mL) value; blue dotted line indicates 95% confidence interval of predictive value.

Fig 2 shows the predicted log 25(OH)D concentration monthly after adjusting for age. The predictive value of vitamin D was obtained using the following model: log(25(OH)D) = 2.092 + 0.009 × age + 0.316 × (month/10)^3^–1.396 × (month/10)^3^ × log(month/10) (*P*<0.001 for all terms in the fitted model, adjusted R^2^ = 0.112). Based on a five-fold CV analysis, the average estimated correlation coefficient between the observed and predicted values in the test sets was 0.322. The maximum level of predicted log 25(OH)D was found in September, and the minimum was found in January.

### Association between serum 25(OH)D level and climate factors

Table 2 shows the association between climate factors and serum 25(OH)D levels as continuous variables using simple and multiple regression analyses. For statistical analyses of normal distribution, 25(OH)D levels were analyzed after logarithmic transformation. In the simple regression analyses, age, monthly mean temperature, accumulated UVA, maximum UVB, and precipitation were correlated with 25(OH)D concentration (all *P* < 0.001). Factors at the 5% significance level in the simple regression model were included in the multiple models. After adjusting for confounding factors (other variables except for oneself) in the multiple regression model, log-transformed 25(OH)D levels increased by 0.009 as age increased by one year and increased by 0.01 as temperature increased by 1°C (P < 0.001). In multiple analyses, age and temperature were significantly associated with serum 25(OH)D concentrations (all *P* < 0.001). Accumulated UVA, maximum UVB, and precipitation were not associated with 25(OH)D levels (P > 0.05).

**Table 2:** Regression analyses of 25(OH)D with log transformation on the variables.

Characteristics	Simple Regression			Multiple Regression		
	B	SE	*P*	B	SE	*P*
Age (year)	0.009	<0.001	<0.001	0.009	0.001	<0.001
Temperature (°C)	0.009	<0.001	<0.001	0.010	0.001	<0.001
Accumulated UVA(MJ/m^2^)	0.095	0.013	<0.001	0.048	0.048	0.309
Maximum of UVB (W/m^2^)	0.839	0.074	<0.001	-0.344	0.283	0.224
Precipitation (mm/day)	0.001	0.001	0.049	<0.001	0.001	0.397
Duration of sunshine (hour/day)	-0.001	0.001	0.256			
Radiation quantity (MJ/m^2^)	<0.001	<0.001	0.701			

^a^Abbreviations: B, beta-coefficient; SE, standard error; UVA, ultraviolet A; UVB, ultraviolet B

**Fig 3:**
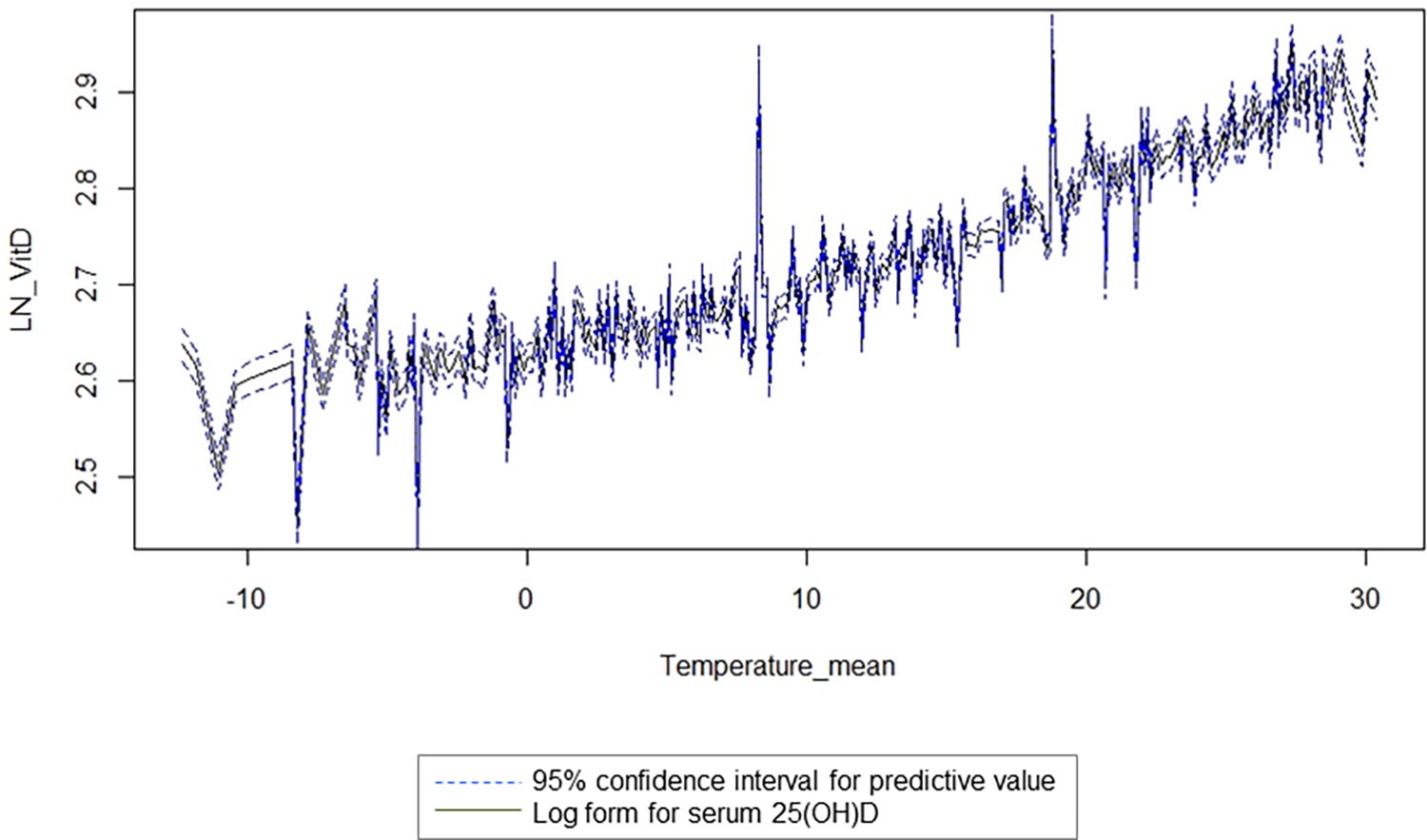
(A) Mean value of the predictive temperature by month. (B) Predictive serum log 25(OH)D concentration according to monthly mean temperature. Black solid line indicates monthly mean value of predicted temperature; blue dotted line indicates 95% confidence interval for predictive value.

Fig 3 shows the predictive level of 25(OH)D with log transformation according to the monthly mean temperature adjusted for age, which was expressed using the following mathematical model: log (25(OH)D) = 2.144 + 0.009 × age + 0.018 × ((temperature + 12.4)/10)^2^ (*P* < 0.001 for all terms in the fitted model; adjusted R^2^ = 0.091). From the five-fold CV analysis, the average estimated correlation coefficient between the observed and predicted value in the test set was 0.299. Serum 25(OH)D level showed an increased tendency between −10 and 30°C according to air temperature.

## Discussion

In this study, serum 25(OH)D levels were significantly associated with age, month, and mean temperature. The serum 25(OH)D concentration was lower in young women than in older women. The proportion of women with adequate 25(OH)D levels was only 14.9% in the age group 20–29 years and 47.0% in the age group 70–79 years. Serum 25(OH)D levels changed monthly. Among the climate factors, monthly mean temperature was associated with serum 25(OH)D concentration. The predictive value of serum 25(OH)D concentration was estimated using a mathematical model based on monthly mean temperature.

In our study, the proportion of adequate 25(OH)D concentration in women was higher in the older age group than the younger age group (based on age 50 years). In addition, highly toxic levels of 25(OH)D have been reported in women aged > 50 years. A previous Korean observational study reported that young women have lower vitamin D levels than older women [3]. When women experience menopause at approximately 50 years of age, they often take nutritional supplements to prevent osteoporosis or fractures [9]. For postmenopausal women, the Korean Society of Bone and Mineral Research recommends the supplementation of calcium and vitamin D [20]. A previous study indicated that younger women engage in fewer outdoor activities than older women [21]. Sunscreen use is more prevalent among the younger generations [22], and has been associated with low vitamin D concentrations [23]. Indoor activity or frequent sunscreen use in young women affects the generation gap in serum 25(OH)D concentration [24]. Increased coffee consumption among young women has been associated with low vitamin D levels in South Korea [25]. Caffeine inhibits calcium metabolism and renal reabsorption, enhancing calcium loss from the kidney or intestine [25]. In an in vitro study, caffeine inhibited osteoblast proliferation and remodeled vitamin D receptor protein expression in osteoblasts [26].

Serum 25(OH)D levels changed according to the month. A predictive model for 25(OH)D with log transformation according to the month was developed using a mathematical formula. However, our results on monthly changes in serum 25(OH)D concentrations are limited to Korean weather conditions. The air temperature can be applied and extended to other conditions. Furthermore, the temperature was the only significant climate factor associated with serum 25(OH)D concentrations in our study. Therefore, we investigated the relationship between serum 25(OH)D concentrations and monthly air temperature using a mathematical formula after adjusting for age. Previous studies have suggested that seasonal variations in serum 25(OH)D levels are associated with the outdoor air temperature [27, 28]. Temperature is directly involved in converting pre-vitamin D3 in the skin [29]. Pre-vitamin D3 undergoes isomerization, converting it into vitamin D3 based on the skin temperature [30]. Human skin temperature is influenced by the outside air temperature [31]. The average monthly air temperature is associated with serum vitamin D concentration [9]. Temperature indirectly affects 25(OH)D by changing the human lifestyle [32]. On a clear and hot day, people prefer outdoor activities with short clothes exposing arms and legs to allow more sunlight for synthesizing vitamin D [8, 33].

The highest temperatures were recorded in July and August. The highest serum 25(OH)D concentration was observed in September. Although vitamin D is synthesized instantly in the skin after exposure to sunlight, the half-life of circulating vitamin D in the body is approximately 2 months [34]. In a previous study, it was observed that the seasonal variation in vitamin D concentration lagged behind that of air temperature by 8 weeks [35]. In our study, the peak points of 25(OH)D concentrations differed between age groups, with the highest concentration in the older age group occurring later than that in the younger age group. Aged human skin is less able to produce pre-vitamin D3 than young epidermis [36]. The production rate of vitamin D3 decreased by 13% per decade of life [37].

This study had some limitations. First, our data did not include the body weight or height of the participants. Fat or body mass index was inversely associated with serum 25(OH)D concentration [4]. Subcutaneous fat tissue deposits 25(OH)D, decreasing the bioavailability of vitamin D to control circulating 25(OH)D [38]. Future studies are needed to analyze the association between obesity and seasonal vitamin D variation. Second, we could not accurately estimate the amount of vitamin D supplementation in enrolled women. Although we excluded women who had a disease or took prescribed medicines that affected serum vitamin D concentration, our study did not analyze individual lifestyle factors, such as dietary habits and nutritional supplements. Third, the power of our mathematical formula for predicting the serum 25(OH)D concentration was relatively low. However, our study is the first large-scale study in women to investigate the relationship among serum vitamin D levels, month, and air temperature. Vitamin D deficiency is associated with various medical conditions, with notable implications for osteoporosis-related fractures. Oral supplementation with vitamin D is essential for maintaining adequate vitamin D levels, especially during winter and in young women [39]. In contrast, high vitamin D levels may be associated with other diseases, including an increased risk of bone fractures [40]. Excessive levels of vitamin D are not recommended for healthy bone metabolism [41]. An adequate strategy for vitamin D supplementation based on the temperature is possible using our formula.

## Conclusion

In women, the 25(OH)D concentration was observed to be lower in the younger generation during winter and at lower air temperatures. A proper monthly strategy to represent the temperature for adequate 25(OH)D levels is necessary to maintain health. Young women should increase their vitamin D supplementation, especially during the cold months. Older women should avoid excessive vitamin D supplementation, especially during hot months. Achieving adequate vitamin D concentrations may require lifestyle changes and oral vitamin D supplementation.
